# Cognitive Enhancement by Transcranial Photobiomodulation Is Associated With Cerebrovascular Oxygenation of the Prefrontal Cortex

**DOI:** 10.3389/fnins.2019.01129

**Published:** 2019-10-18

**Authors:** Emma Holmes, Douglas W. Barrett, Celeste L. Saucedo, Patrick O’Connor, Hanli Liu, F. Gonzalez-Lima

**Affiliations:** ^1^Department of Psychology, Institute for Neuroscience, University of Texas at Austin, Austin, TX, United States; ^2^Department of Bioengineering, University of Texas at Arlington, Arlington, TX, United States

**Keywords:** photobiomodulation, near-infrared spectroscopy, prefrontal cortex, cognitive enhancement, cerebrovascular oxygenation

## Abstract

Transcranial infrared laser stimulation (TILS) is a novel, safe, non-invasive method of brain photobiomodulation. Laser stimulation of the human prefrontal cortex causes cognitive enhancement. To investigate the hemodynamic effects in prefrontal cortex by which this cognitive enhancement occurs, we used functional near-infrared spectroscopy (fNIRS), which is a safe, non-invasive method of monitoring hemodynamics. We measured concentration changes in oxygenated and deoxygenated hemoglobin, total hemoglobin and differential effects in 18 healthy adults during sustained attention and working memory performance, before and after laser of the right prefrontal cortex. We also measured 16 sham controls without photobiomodulation. fNIRS revealed large effects on prefrontal oxygenation during cognitive enhancement post-laser and provided the first demonstration that cognitive enhancement by transcranial photobiomodulation is associated with cerebrovascular oxygenation of the prefrontal cortex. Sham control data served to rule out that the laser effects were due to pre-post task repetition or other non-specific effects. A laser-fNIRS combination may be useful to stimulate and monitor cerebrovascular oxygenation associated with neurocognitive enhancement in healthy individuals and in those with prefrontal hypometabolism, such as in cognitive aging, dementia and many neuropsychiatric disorders.

## Introduction

Photobiomodulation techniques using lasers or LEDs in the far-red to near-infrared spectrum, also called low-level light/laser therapy, have been widely used for wound healing, musculoskeletal pain, arthritis, and other conditions (Brosseau et al., 2004; Hopkins et al., 2004; Cotler et al., 2015). Brain cells are highly dependent on oxygen supply for energy metabolism. Various forms of photobiomodulation of the nervous system are currently being tested for applications in aging-related neurocognitive decline (Vargas et al., 2017), depression and anxiety (Disner et al., 2016; Caldieraro and Cassano, 2019) as well as various neurological disorders (Naeser and Hamblin, 2011; Holanda et al., 2017). Recently, transcranial infrared laser stimulation (TILS) by 1064-nm laser has been introduced as a possible means of human cognitive enhancement (Barrett and Gonzalez-Lima, 2013; Gonzalez-Lima and Barrett, 2014; Hwang et al., 2016; Blanco et al., 2017a,b).

This experiment attempted to elucidate the hemodynamic effects of TILS of the human prefrontal cortex at the wavelength, energy and power density used in previous cognitive augmentation studies. The goal was to provide further support for the proposed mechanism of cerebral oxygenation of this treatment (Tian et al., 2016; Wang et al., 2017) by gathering real-time hemodynamic data from around the prefrontal site of laser administration during cognitive performance. A better understanding of the hemodynamic effects of this new method for cognitive enhancement may allow progress toward its use as a clinical treatment.

Photobiomodulation techniques influence mitochondrial respiration and promote beneficial cellular functions in multiple tissues, including nervous tissue (Eells et al., 2004; Wong-Riley et al., 2005). It has been suggested that brain photobiomodulation has a photonic action on cytochrome-c-oxidase (CCO) that leads to hemodynamic effects (Rojas and Gonzalez-Lima, 2011; Gonzalez-Lima and Barrett, 2014). Near infrared photons pass through the forehead (Tedford et al., 2015) to affect the functioning of tissue on the cortical surface, upregulating the enzymatic activity of CCO, the final and rate-limiting step in the mitochondrial electron transport chain. CCO is responsible for oxygen consumption at the cellular level, which is vital in oxidative phosphorylation for the production of ATP, the primary source of biochemical energy in the body. Mitochondrial respiration driven by neural activation leads to higher demand for energy production that needs to be maintained by increasing the supply of oxygenated blood from the circulation. Alternatively, the laser stimulation might produce transcranial effects due to the heat produced by the laser or the LEDs (Ito et al., 2000). However, it has recently been confirmed that the effects of TILS are due to the effect of the photons themselves that photo-oxidize CCO to a conformation that has more affinity for oxygen consumption, rather than the effects of heat on increasing metabolism or circulation (Wang et al., 2018). This further supports the hypothesis that photons from TILS are acting by upregulating CCO and therefore having an enhancing effect on mitochondrial energy metabolism and cerebral hemodynamics (Wang et al., 2017).

Functional near-infrared spectroscopy (fNIRS) is an optical method that usually maps the transient hemodynamic responses in the cerebral cortex during functional tasks, including cognitive tasks such as working memory (Tian et al., 2014). However, a different application of fNIRS with two source-detector channels served to investigate longer time (minutes) cerebral hemodynamic effects during TILS (Tian et al., 2016). In our present application of fNIRS, we combined cognitive tasks with 20 source-detector channels to measure longer time effects before and after TILS. The head was fitted with a close cap covered in grommets with positions consistent with the 10–20 EEG mapping system. These grommets served as housings for a montage of 20 channels of optical emitters and detectors, spaced 3-cm apart. In dual-wavelength fNIRS, emitters serve as sources for two wavelengths of light, each on either side of the isosbestic point of hemoglobin at 810 nm. One wavelength is primarily absorbed by oxygenated hemoglobin (HbO_2_), whereas the other wavelength is primarily absorbed by deoxygenated hemoglobin (Hb). The detectors use fiber optic cables to extract photons not absorbed by hemoglobin and transmit them into the spectroscopy system. The computational system is then able to determine the relative change (Δ) in concentrations of oxygenated hemoglobin [HbO_2_] and deoxygenated hemoglobin [Hb]. Dual-wavelength fNIRS has already been used to show the longer time effects of TILS on cerebral oxygenation in humans who were not performing cognitive tasks (Tian et al., 2016).

Previously, TILS using a 1064 nm wavelength was used to augment cognitive functioning and behavioral data revealed statistically significant improvements in performance on the psychomotor vigilance task (PVT) and the delayed match-to-sample task (DMS) in healthy young adults (Barrett and Gonzalez-Lima, 2013; Hwang et al., 2016). The purpose of the present study was to use fNIRS to map hemodynamic responses during cognitive activation before and after TILS to the prefrontal cortex, under conditions that included these cognitive tasks. A greater understanding of the physiological effects of TILS on prefrontal cognitive enhancement may facilitate its use in clinical settings.

## Materials and Methods

### Subjects

A total of 34 healthy adult participants (16 male, 18 female; average age: 31, standard error: 2.5) were recruited from the University of Texas at Austin. The 18 experimental group participants (9 male, 9 female) received full laser stimulation and completed all tasks using a within-subject control design. They performed the cognitive tasks before and after TILS, with concomitant fNIRS recordings, to reflect the hemodynamic effects of TILS on cognitive performance. Another 16 participants (7 male, 9 female) were matched blind to treatment as sham controls without photobiomodulation (TILS procedure used with light off). Cognitive data was measured in seven of the sham participants (3 male, 4 female) as previously described (Barrett and Gonzalez-Lima, 2013). Hemodynamic data was measured in nine of the sham participants (4 male, 5 female) as previously described (Wang et al., 2017). The protocol was approved by the University of Texas at Austin’s Institutional Review Board and complied with all applicable federal and NIH guidelines. All subjects gave written informed consent in accordance with the Declaration of Helsinki.

### Functional Near-Infrared Spectroscopy

**FIGURE 1:**
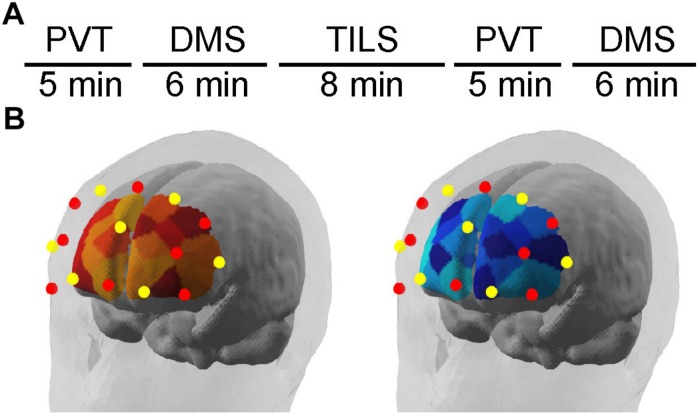
**(A)** Experimental design timeline to obtain the fNIRS results during cognitive testing with the psychomotor vigilance task (PVT) and delayed match-to-sample task (DMS), before and after transcranial infrared laser stimulation (TILS). **(B)** Illustrates the position of fNIRS channels relative to brain areas and the separate monitoring of changes in [Hb] and [HbO_2_] using a dual-wavelength fNIRS system. Schematic location of fNIRS map of frontal cortex hemodynamics as illustrated by the NirSTAR software (NIRx Medical Technologies, LLC; Minneapolis, MN, United States). Red dots represent emitters; yellow dots represent detectors. Relative changes in oxygenated hemoglobin (left) and deoxygenated hemoglobin (right) were quantified as illustrated in the brain schematic diagrams.

**FIGURE 2:**
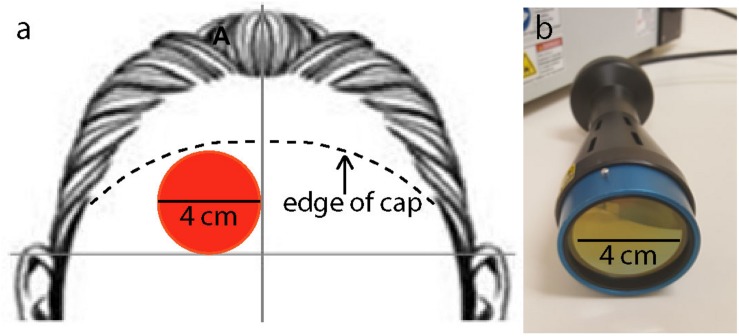
**(a)** Location of laser stimulation treatment on the right forehead: 4-cm diameter site (red circle) in the corner lateral to the midline of the forehead and superior to a horizontal line above the two ears. Laser treatment location was not moved throughout the full 8 min of treatment. The figure also illustrates where the approximate edge of the cap was relative to the 4-cm laser spot (segmented lines). **(b)** Laser instrument used to administer the laser treatment with a collimation optical device of 4 cm aperture diameter.

Figure 1A illustrates the experimental timeline. Figure 1B illustrates the position of fNIRS channels relative to brain areas and the separate collection of measurements of [Hb] and [HbO_2_]. The fNIRS system used for this experiment was a NIRScout Standard system outfitted with LED light sources and Avalanche Photodiode Detectors (the most sensitive light detectors offered by NIRx Medical Technologies, LLC; Minneapolis, MN, United States). An fNIRS cap was fitted based on the circumference of the head (ranging 54–58 cm). Using the cotton end on a stick, the hair was moved out of the way within the grommets to be used in the montage. The caps were then fitted with a montage of eight emitters and seven detectors arrayed on the head above the frontal bone. Figure 2 illustrates where the approximate edge of the cap was relative to the 4-cm laser spot. An overcap was then placed on top of the full montage to reduce extraneous effects due to ambient light. The fNIRS system was connected to the NirSTAR operating program on a separate computer. The system was then calibrated and automatically adjusted until there was a stable signal detected within each emitter-detector paired channel. During recording epochs, the system recorded at a sampling rate of 7.8125 Hz, or about eight measurements per second. Each emitter/detector pair corresponded to one channel, with 20 channels in total, with 3-cm source-detector distances.

After calibration, the fNIRS system collected data for 2 min while the participant was inactive and maintained normal respiratory and blinking rates. These data provided the zero baseline from which changes in the hemoglobin dependent variables were calculated as described below. The participant then began the PVT task while the fNIRS system took measurements throughout the task (5 min). The same was done during the administration of the DMS task (6 min). The fNIRS system was not gathering data during the laser treatment (8 min), to prevent the laser from possibly interfering with the optodes; however, the placement of the cap, emitters, detectors, and overcap remained unchanged throughout administration of the laser. The same procedure for the PVT and DMS was repeated post-TILS. The post-TILS tasks and fNIRS started within 2 min after laser irradiation.

Following the post-treatment tasks, the fNIRS system was turned off and the apparatus was removed from the participant. The fNIRS system automatically used the modified Beer-Lambert law in order to calculate the relative concentrations of oxygenated hemoglobin [HbO_2_] and deoxygenated hemoglobin [Hb].

### Transcranial Infrared Laser Stimulation

This study used a collimated laser diode with a wavelength of 1064 nm supplied by Cell Gen Therapeutics, LLC (Model CG-5000 laser, HD Laser Center; Dallas, TX, United States). The wavelength used in this and other studies from this lab is longer than is typically used (Rojas and Gonzalez-Lima, 2011). This longer wavelength was used to improve transmission through the cortical surface and to reduce light scattering as compared to shorter wavelengths. Light scattering is the dominant light-tissue interaction, but scattering decreases with longer wavelengths (Jacques, 2013).

This device has been cleared as safe for human use by the Food and Drug Administration (FDA) for indications of muscle or joint pain, improving circulation, improving muscle aches, and wound healing. This device has not been evaluated or approved by the FDA for the specific uses in this study. The power density of the treatment was 250 mW/cm^2^, and the energy density of the treatment was 120 J/cm^2^. These parameters were chosen because they showed significant cognitive benefits in our previous studies (Barrett and Gonzalez-Lima, 2013; Blanco et al., 2017a; Vargas et al., 2017). Approximately 1–2% of the laser treatment applied to the forehead at a wavelength of 1064 nm passes through the frontal bone (Barrett and Gonzalez-Lima, 2013); therefore approximately 1.2–2.4 J/cm^2^ reaches the cortical surface. The aperture of the laser extended in a 4 cm diameter circle. Figure 2b illustrates the laser device and aperture used in this study.

All participants received TILS once, on the day of cognitive testing. The laser treatment was applied to the right side of the forehead, targeting the right prefrontal cortex, which is the brain region showing the most effective cognitive-enhancing effect in our PVT and DMS studies (Barrett and Gonzalez-Lima, 2013; Hwang et al., 2016; Vargas et al., 2017). Previous studies in the lab have alternated placement of laser application between different sites in the forehead; however, because of constraints due to the fNIRS cap, there was only room on the participant’s right forehead for treatment in a single site. Figure 2a illustrates the forehead placement (target) of the laser stimulation. Concerning the 10–20 electrode mapping system used in EEG, the laser aperture was just below the Fp2 point (right frontal pole). The laser treatment was delivered for 8 min from a distance of approximately 10 inches.

The researcher and participant were locked in a non-reflective room with a sign on the outer door indicating that the laser was in use. All participants and lab personnel present wore protective eyewear guarding against the 1064-nm wavelength (900–1000 nm: 5 +, 1000–2400 nm: 7+; 2900–10600 nm: 7+) in accordance with safety operating procedures for this machine, and the participants had their eyes closed at all times while the laser treatment was being administered.

Laser stimulation parameters

Center wavelength (nm): 1064

Spectral bandwidth (nm): 5 (FWHM)

Operating mode: CW

Average radiant power (mW): 3400

Polarization: No (after fiber transmission)

Aperture diameter (cm): 4

Irradiance at aperture (mW/cm^2^): 250

Beam divergence: Near zero (well-collimated beam)

Beam shape: Circular

Beam profile: Top Hat

Beam spot size at forehead target (cm^2^): 13.6

Irradiance at forehead target (mW/cm^2^): 250

Exposure duration (s): 480

Radiant exposure (J/cm^2^): 120

Radiant energy (J): 1632

Number of points irradiated: One

Forehead area irradiated (cm^2^): 13.6

Delivery mode: Non-contact; 10 inches distance of the beam source to the forehead target

Number and frequency of treatment sessions: One session; delivered in 8 min

Total radiant energy (J): 1632

### Cognitive Testing

The PVT was administered to participants as the first cognitive task to measure reaction times before and after TILS. At both time points, the fNIRS system was run continuously during the task. The PVT is a computer-based task originally developed by Dinges and Powell (1985) as a visual reaction time task. This task was originally used to test reaction time under sustained operations; however, it has now been modified to test general sustained attention and vigilance in healthy individuals. This task has been shown to be a reliable indicator of frontal cortex function (Drummond et al., 2005). In this task, participants attend to a white fixation cross in the middle of a black background. After a short period of time, the fixation cross disappears and there is a variable period of delay where the screen remains black. After the delay, white numbers begin counting up in the middle of the screen in milliseconds. The participants are instructed to press the space bar as soon as they see the numbers appear. The counter briefly stops to display the final reaction time before moving on to the next trial. If participants press the space bar before the appearance of the numbers, a message of “Too Soon!” is briefly displayed on the screen. If the participants take too long to respond (greater than 500 ms), the trial is recorded as a lapse in attention. The PVT consisted of 40 trials, and the delay period between the presentation of the fixation cross and the stimulus varied pseudo randomly between 2, 4, and 6 s. The duration of the PVT was approximately 5 min. The two dependent variables recorded by the computer are reaction time (in msec) and the number of “correct” trials. If the participant responded too soon (before the onset of the stimulus) or too late (after 500 ms have elapsed), the trial was coded as incorrect.

The DMS was administered to participants directly following their completion of the PVT to measure working memory both pre-treatment and post-treatment. Performance on this working memory task has previously been shown to be mediated by a frontoparietal network (Nieder and Miller, 2004). In this task, participants are presented with a 6 × 6 grid of red and yellow squares in a random pattern presented on a plain gray background. A delay of 6 s follows, during which only the gray background is shown. After the delay period, two 6 × 6 grids are presented side by side. One grid (the “match”) is identical to the original grid, and the second grid (the “foil”) is slightly different. The participant must respond with either the left or right SHIFT key to indicate which grid is the match. Following the choice, either “Correct” or “Incorrect” are briefly displayed onscreen based on the participant’s choice. If the participant does not answer within 4 s, the trial times out and the feedback given is “Timeout.” The DMS consisted of 30 trials, and the duration of the DMS was approximately 6 min. The two dependent variables recorded by the computer were response time (in ms) during the choice part of the task and the number of correct trials.

### Data Analysis

The data from the optical signal obtained from the fNIRS apparatus was processed using the software NirsLAB (v2017.6) provided by NIRx Optical Neuroimaging. All epochs/channels were checked to ensure that at no time there were any channels saturated; no saturated intervals were found. Each recording epoch was checked for data quality in all 20 channels, and if the gain provided by the initial calibration step was not within the recommended range, that data was discarded. Out of 20 channels × 18 participants = 360 time series of data for all participants, eight were removed from the analysis. The data was then processed using a band-pass filter, with a low cut-off frequency of 0.01 Hz (to remove the widely observed slow baseline drift) and a high cut-off frequency of 0.2 Hz (to remove electronic noise and fast-oscillating cardiac waves) (Tian et al., 2010; Piper et al., 2014). The modified Beer-Lambert Law was used to calculate changes in oxygenated hemoglobin concentration (Δ[HbO_2_]) and deoxygenated hemoglobin concentration (Δ[Hb]) relative to the initial baseline set as zero (Tian et al., 2016; Wang et al., 2016). Total hemoglobin (Δ[HbT]) was calculated with a sum (Δ[HbO_2_] + Δ[Hb]) and differential concentrations of hemoglobin (Δ[HbD]) were calculated using a difference score (Δ[HbD] = Δ[HbO_2_] – Δ[Hb]) as in Tian et al. (2016) and Wang et al. (2017). This value (Δ[HbD]) is an indicator of changes in tissue oxygen saturation (Tian et al., 2016) which is closely correlated with changes in cerebral blood flow (Wang et al., 2017). These values were calculated and averaged for each recording epoch for each subject. The fNIRS system gives only relative values (not absolute values) of these measures; therefore, the first fNIRS readings (taken before the first PVT session) were used as a zero-point by subtracting those values for each subject/channel from each subsequent measurement, to standardize the data and create a common zero-point baseline shared by all subjects.

To evaluate the effect of the TILS treatment, all 20 channels were averaged together to create an index of global cerebral blood flow (across the entire range of frontal cortex sampled, including both hemispheres) for each subject/epoch. These “all-channels” averages for the values of HbO_2_, Hb, HbT, and HbD were compared between pre-TILS and post-TILS measures using paired *t*-tests with a two-tailed level of significance with *p* ≤ 0.01.

For each subject’s cognitive performance in both sessions of the PVT and DMS, an overall cognitive score was calculated, incorporating both speed (reaction time) and accuracy (number of correct responses), using the rate correct score or RCS (Woltz and Was, 2006). This overall cognitive score was equal to the number of correct responses divided by the sum of all reaction times. The PVT and DMS behavioral results were analyzed with paired *t*-tests comparing performance on these tasks before and after TILS with a two-tailed level of significance with *p* ≤ 0.01.

## Results

### Cognitive Effects of TILS and Sham

**FIGURE 3:**
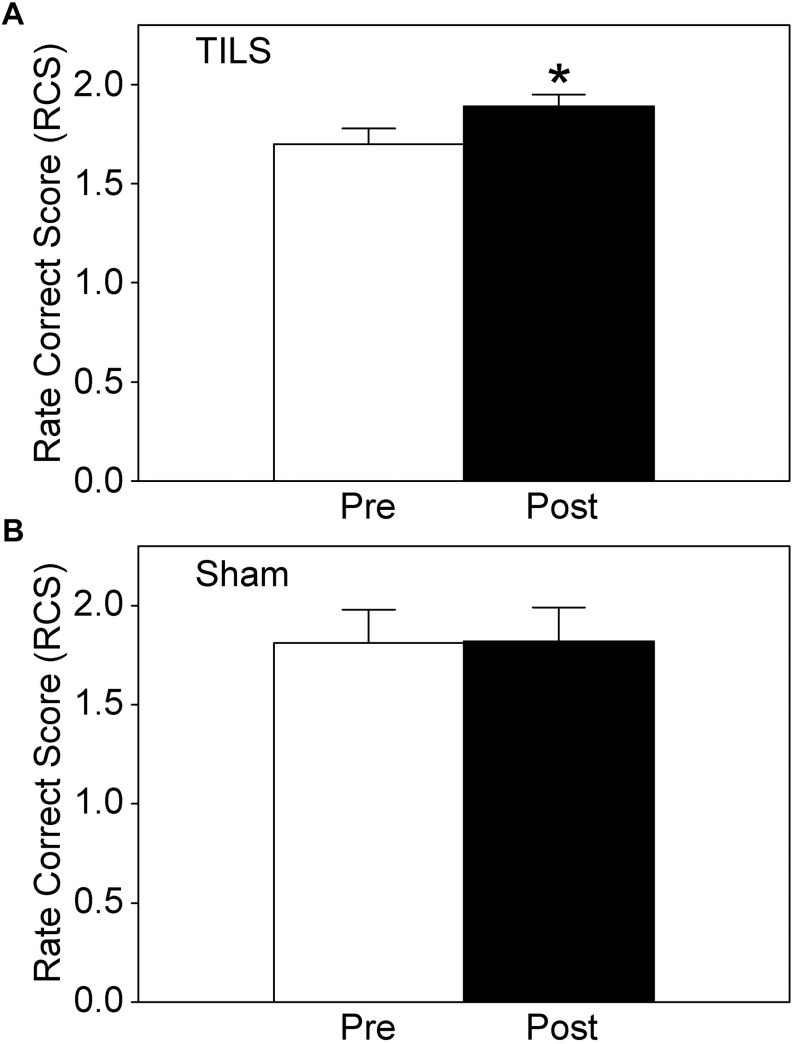
Rate correct score (RCS) for cognitive performance before and after TILS **(A)** and Sham **(B)**. Mean ± S.E., ^∗^ = Significant mean difference between Pre- vs. Post- TILS scores, *p* ≤ 0.01. ^∗^No randomization occurred for sham, since we used a within-subject design in which the same subjects are their own control by directly comparing pre- vs post- measures statistically. There were no significant pre-post differences in the sham subjects.

Cognitive results from the PVT and DMS were obtained before and after TILS and sham. As expected, overall cognitive processing improved after TILS, as indicated by the significantly higher rate correct score (Figure 3A), whereas there were no significant differences after sham (Figure 3B). This score reflects the speed and accuracy of cognitive processing. The overall rate correct score effect size (Cohen’s *d*) after TILS was |*d*| = 0.62, indicating a medium effect size, given that |*d*| < 0.2 = small effect; 0.2 < |*d*| < 0.8 = medium effect; |*d*| > 0.8 = large effect (Cohen, 1988).

Table 1 summarizes the details of all the individual scores for TILS and sham. On average, the DMS contributed stronger effect sizes as compared to the PVT. Average performance in the DMS improved after TILS, with significant improvements in both number of correct answers and memory retrieval time (i.e., time to make a choice between the match and foil grids) as assessed by paired *t*-tests. Performance on the PVT tended to improve, in terms of faster reaction times after the TILS treatment, as well as an increase in the number of correct trials (where a correct trial is defined as neither a “too-fast” trial nor a “lapse” with a reaction time greater than 500 ms), but this improvement was not significant at *p* ≤ 0.01.

**TABLE 1:** Results of cognitive tests before (Pre) and after (Post) TILS and Sham.

**Dependent variable**	**Pre-TILS**	**Post-TILS**			
					
	**Mean ± S.E.**	**Mean ± S.E.**	***t*-Score**	***p*-value**	**Cohen’s *d***
**A. LASER**					
PVT reaction time (ms)	331.6 ± 6.10	329.2 ± 6.73	–0.53	0.60	0.09
PVT correct trials (out of 40)	38.5 ± 0.43	39.3 ± 0.23	1.74	0.10	0.52
DMS response time (s)^∗^	2.03 ± 0.07	1.83 ± 0.07	–3.17	0.01	0.64
DMS correct trials (out of 30)^∗^	22.3 ± 0.82	24.7 ± 0.61	2.85	0.01	0.73

**Dependent variable**	**Pre-Sham**	**Post-Sham**			
					
	**Mean ± S.E.**	**Mean ± S.E.**	***t*-Score**	***p*-value**	**Cohen’s *d***

**B. SHAM**					
PVT reaction time (ms)	316.1 ± 14.9	326.4 ± 20.6	1.57	0.17	0.22
PVT correct trials (out of 40)	39.0 ± 0.53	38.4 ± 0.57	–0.83	0.44	0.39
DMS response time (s)	2.1 ± 0.14	2.0 ± 0.12	–0.67	0.53	0.00
DMS correct trials (out of 30)	22.4 ± 1.38	23.0 ± 1.07	0.40	0.70	0.18

*^∗^No randomization occurred for sham, since we used a within-subject design in which the same subjects are their own control by directly comparing pre- vs. post- measures statistically. There were no significant pre-post differences in the sham subjects (Mean ± S.E., ^∗^ = Significant mean difference between Pre- vs. Post- TILS scores, *p* ≤ 0.01).*

Sham cognitive data analyses showed no significant differences in any of the pre-post comparisons, using the same methods as in the TILS group (Table 1). As expected from two previous sham-controlled studies using the same PVT and DMS tasks (Barrett and Gonzalez-Lima, 2013; Hwang et al., 2016), cognitive processing did not improve significantly after sham, as shown by nearly identical pre-post rate correct scores. Mean ±SE for sham, pre: 1.81 ±0.17. Mean ±SE for sham, post: 1.82 ±0.17. The value of *t* is 0.077143. The value of *p* is 0.94102. The result is *not* significant at *p* ≤ 0.05.

### Hemodynamic Effects of TILS and Sham

There was a significant effect of TILS on three of the dependent variables: Δ[HbO_2_], Δ[HbT], and Δ[HbD] all showed significant increases at *p* ≤ 0.01 after TILS but not after sham. Deoxygenated hemoglobin Δ[Hb] did not show a significant difference after TILS. The significant hemodynamic effects of TILS showed large effect sizes (|*d*| = 0.94–1.02). Tables 2, 3 shows means ± standard errors and the values of *t* and *p* across all channels during the PVT and DMS pre-post comparisons, respectively. No single channel was driving the significant effect, as all channels were highly co-linear with each other. There were no other significant differences between PVT and DMS besides the TILS main effect on the hemodynamic response.

**TABLE 2:** Cerebral hemodynamic changes before (Pre) and after (Post) TILS averaged across all 20 channels during the PVT.

**PVT**	**Pre-TILS**	**Post-TILS**			
					
	**Mean ± S.E.**	**Mean ± S.E.**	***t*-score**	***p*-value**	**Cohen’s *d***
HbO_2_^∗^	0.0412 ± 0.0194	0.2199 ± 0.0489	4.28	0.001	0.99
Hb	−0.0095 ± 0.0027	−0.0066 ± 0.0062	0.45	0.665	0.14
HbT^∗^	0.0316 ± 0.0191	0.2134 ± 0.0478	4.27	0.001	1.02
HbD^∗^	0.0507 ± 0.0201	0.2265 ± 0.0507	4.18	0.002	0.95

*(μM; Mean ± S.E., ^∗^ = Post-TILS scores significantly greater than Pre-TILS scores, *p* ≤ 0.01).*

**TABLE 3:** Cerebral hemodynamic changes before (Pre) and after (Post) TILS averaged across all 20 channels during the DMS.

**DMS**	**Pre-TILS**	**Post-TILS**			
					
	**Mean ± S.E.**	**Mean ± S.E.**	***t*-score**	***p*-value**	**Cohen’s *d***
HbO_2_^∗^	0.0390 ± 0.0201	0.2162 ± 0.0494	4.17	0.001	0.97
Hb	−0.0095 ± 0.0029	−0.0076 ± 0.0061	0.30	0.768	0.10
HbT^∗^	0.0295 ± 0.0196	0.2086 ± 0.0484	4.14	0.001	1.00
HbD^∗^	0.0485 ± 0.0210	0.2237 ± 0.0512	4.11	0.001	0.94

*(μM; Mean ± S.E., ^∗^ = Post-TILS scores significantly greater than Pre-TILS scores, *p* ≤ 0.01).*

Sham hemodynamic data analyses showed no significant differences in any of the pre-post comparisons. As expected from a previous hemodynamic study of TILS and sham effects (Wang et al., 2017), none of the hemodynamic measures changed significantly after sham. Δ[HbO_2_]: The value of *t* is 1.7928. The value of *p* is 0.11076. The result is *not* significant at *p* ≤ 0.05. Δ[Hb]: The value of *t* is 0.945994. The value of *p* is 0.37184. The result is *not* significant at *p* < 0.05. Δ[HbT]: The value of *t* is 2.272126. The value of *p* is 0.05772. The result is *not* significant at *p* < 0.05. Δ[HbD]: The value of *t* is 1.351153. The value of *p* is 0.21361. The result is *not* significant at *p* < 0.05.

Since significant effects were found only after TILS, we further analyzed all the TILS data in terms of temporal and spatial effects as described below. No further analysis of the negative sham data was done because there were no significant hemodynamic sham effects to analyze.

### Temporal and Spatial Hemodynamic Effects of TILS

**FIGURE 4:**
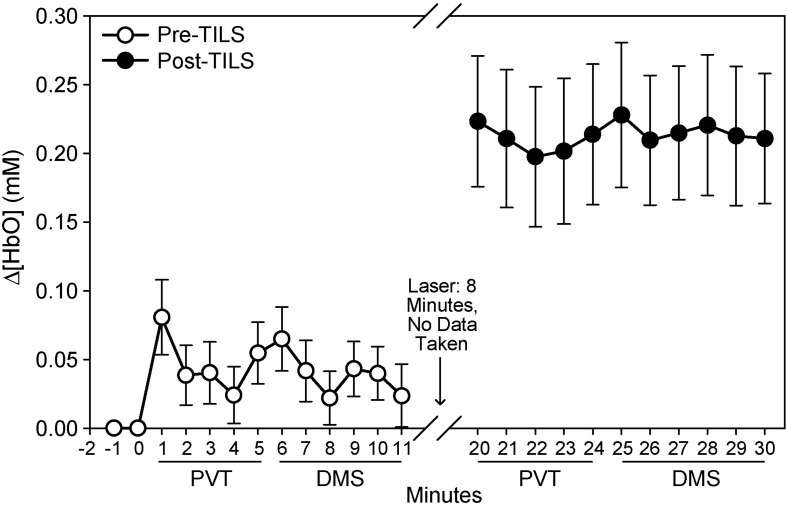
Temporal sequence of changes in oxygenated hemoglobin (μM), pre- and post-TILS during cognitive processing in the PVT and DMS tasks. Mean ± S.E.

**FIGURE 5:**
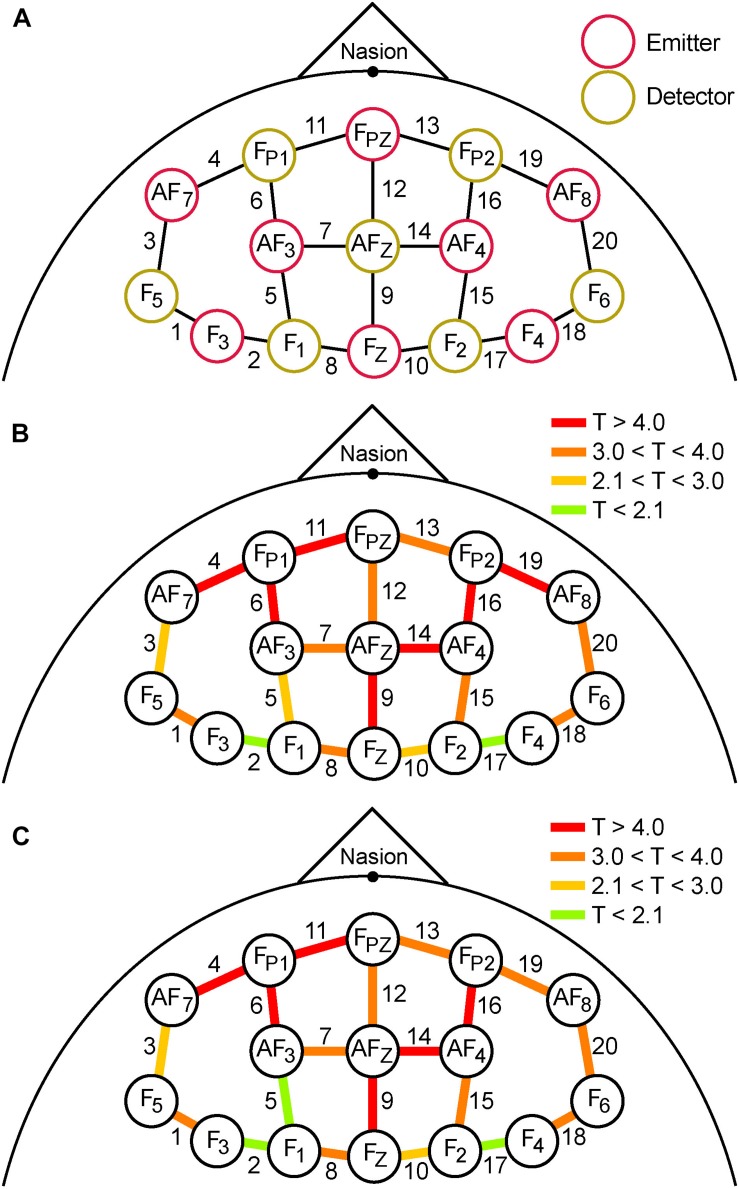
**(A)** Schematic spatial layout of the frontal montage used for fNIRS recording. Red and yellow circles correspond to emitters and detectors, respectively. The labels inside the circles correspond to the 10–20 EEG system locations used for each emitter and detector. Each adjacent emitter/detector pair formed a channel that gathered fNIRS data (20 labeled lines). **(B)** Colored lines represent the magnitude of T-scores from the analysis of mean differences between pre- and post-TILS PVT epochs. **(C)** The magnitude of T-scores comparing the pre- and post-TILS DMS epochs. The affected channels are almost identical, with two exceptions (channels 5 and 19).

The effects of TILS on the fNIRS signal are depicted in Figures 4, 5, in terms of the temporal and spatial effects of TILS, respectively. The level of oxygenated hemoglobin rose significantly after TILS and maintained a higher level throughout the post-TILS period (Figure 4). We made an additional statistical comparison because the relative temporal pattern of Δ[HbO_2_] during PVT appeared to change from before to after TILS regardless of baseline, and we determined whether this was a significant difference. We verified that this was not a significant difference. We performed repeated measures ANOVA on Δ[HbO_2_] during the 5 min of PVT, minute by minute, pre-TILS vs. post-TILS. Therefore, there were two within-subject variables. We were interested in the interaction between pre-post and minutes. In other words, was the shape of the curve during those 5 min statistically different between pre-TILS and post-TILS. That interaction was not significant: Pre-post × Minutes: *F*(4,68) = 0.991, *p* = 0.419.

Comparing differences between pre- and post-TILS in terms of spatial effects, the anterior channels in the fNIRS frontal montage (Figure 5A) registered higher levels of oxygenated hemoglobin than the posterior channels during cognitive processing of PVT (Figure 5B) and DMS (Figure 5C). To verify that the significant effects within individual channels was not due to multiple statistical comparisons, the “Sharper Bonferroni” correction was used (Hochberg, 1988), in which *p*-values are corrected for multiple comparisons using multiplication according to ranked order; with 20 comparisons (one for each channel), the strongest effect is multiplied by 20, the next-strongest by 19, etc. The channels that still showed significant pre-to-post-TILS increases in HbO_2_ during both the PVT and DMS epochs, with corrected values of *p* ≤ 0.01, were channels 4, 6, 9, 11, 14, 16, and 19. These are also the channels with the highest *T*-values (red in Figures 5B,C) in the anterior part of the frontal montage.

## Discussion

This study is the first to demonstrate that cognitive enhancement by transcranial photobiomodulation is associated with cerebrovascular oxygenation of the prefrontal cortex. The control group (sham) behavioral data and hemodynamic data (with no photobiomodulation) showed no significant differences in any of the pre-post comparisons. These sham control data served to rule out that the laser effects were due to pre-post task repetition or other non-specific effects.

The main finding was the highly significant hemodynamic effect of TILS on Δ[HbO_2_], Δ[HbT], and Δ[HbD] on the anterior frontal cortex during cognitive processing. The laser treatment resulted in a large increase in oxygenated hemoglobin in the anterior frontal region measured by the fNIRS apparatus, corresponding to the prefrontal cortex region engaged during the PVT and DMS tasks (Nieder and Miller, 2004; Drummond et al., 2005). This large hemodynamic response was about five times greater than in the pre-TILS condition and it was sustained for more than 10 min of cognitive processing after TILS.

The concern that heating might cause non-specific fNIRS changes rather than the neurophotonic stimulation has been addressed directly in our recently published paper (Wang et al., 2018) that compares the effects of heating vs. TILS on the cerebral hemodynamic responses. The results showed that thermal stimulation of the forehead does not produce the effects of TILS on Δ[HbO_2_], Δ[HbT], and Δ[HbD] (e.g., Figure 6 of Wang et al., 2018). These results served to rule out the concern that heating from our cold laser might be responsible for any signal changes that could explain the cerebral hemodynamic effects measured by fNIRS.

The improvement in overall cognitive performance following TILS was much smaller as compared to the hemodynamic response. To interpret this finding we considered the results from another previous study (Wang et al., 2017) comparing hemodynamic effects on superficial tissues and brain to address how reflective the fNIRS measure is of brain changes and how much is this measure contaminated by changes in oxygenation from superficial tissues. The results showed that laser-induced superficial skin and tissue changes (measured at 1.5 cm source-detector distance) are about 5% above baseline whereas brain changes (measured at 3 cm source-detector distance) are about 20% when compared using metabolic/hemodynamic ratios (e.g., Figure 5 of Wang et al., 2017). Therefore, TILS-induced cerebral hemodynamic changes do not appear to receive a heavy contribution from superficial tissues that could account for our findings that fNIRS brain changes are much greater than changes in cognition. This is consistent with the general finding that hemodynamic responses are several times greater than observable cognitive differences and levels of tissue oxygen consumption (Fox, 2012; Beishon et al., 2018).

The behavioral sham data comparing pre-post tasks without TILS served to confirm the lack of learning/placebo effects for repeated testing of our specific DMS and PVT tasks, and replicated the same sham findings from our published studies. The cognitive effects are consistent with our previous two studies in which we compared TILS with sham/placebo groups in the same PVT and DMS tasks (Barrett and Gonzalez-Lima, 2013; Hwang et al., 2016). Indeed, pre-post testing of these PVT and DMS tasks in a single session without laser stimulation do not lead to improved cognitive scores (Barrett and Gonzalez-Lima, 2013). Therefore, placebo or practice effects cannot explain the cognitive improvement produced by TILS. The fact that the improvement in PVT performance was not statistically significant at the *p* ≤ 0.01 level may be based on the single treatment given because repeated TILS leads to a significant increase in PVT scores (Vargas et al., 2017). In addition, we have previously found significant improvements caused by TILS as compared to sham in other prefrontal-dependent cognitive tasks, such as the Wisconsin card sorting task (Blanco et al., 2017a) and rule-based category learning (Blanco et al., 2017b).

The main limitation of the study was the lack of data acquired during the TILS administration, which was implemented to avoid the possibility of damage to the fNIRS system’s photodetectors by the laser. Other fNIRS systems contain a shutter system to maintain the recording during TILS without damaging the photodetectors. Also, the system only returns the relative changes in HbO_2_ and Hb, with no absolute “zero” point, making it difficult to compare across subjects; this issue necessitated the use of the initial recording period as a zero-point by subtracting it from subsequent data points within the time series.

In the future, an event-related design will be implemented, using event markers during the different phases of the PVT and DMS to examine hemodynamic changes within each stage of the task (e.g., encoding vs. maintenance vs. retrieval of the visuospatial memory during the DMS) as done in our previous fNIRS study of working memory in post-traumatic stress disorder (Tian et al., 2014). Prefrontal neurons associated with working memory are selectively active during the maintenance phase of the DMS (Sawaguchi and Yamane, 1999); synchronizing the different phases of the task with simultaneous fNIRS readings may yield more task-specific temporal results.

Transcranial infrared laser stimulation may be useful as a safe, non-invasive, non-pharmacological and cost-effective approach to increasing cognitive function. The scope of the hemodynamic effect of TILS on human brains may be monitored in real time using fNIRS. This combined TILS-fNIRS system could be a tool for titrating the dose of laser treatment for each individual based on their own particular cerebrovascular oxygenation response. Additionally, this system might help identify good candidates for therapeutic intervention with laser stimulation prior to presentation of symptoms based on hypofrontality. In the future, this laser treatment could be used for populations presenting with prefrontal hypometabolism, such as in cognitive aging, mild cognitive impairment, Alzheimer’s disease, and many other neurological and psychiatric conditions that lead to cognitive decline (Rojas and Gonzalez-Lima, 2013).

## Conclusion

In conclusion, TILS at 1064 nm wavelength enhanced cognitive performance and fNIRS response in human prefrontal cortex. Together the results of this study and our previous studies suggest that TILS is a form of photobiomodulation that can successfully augment cerebrovascular oxygenation and thereby improve human cognitive brain functions.

## Data Availability Statement

The raw data supporting the conclusion of this manuscript will be made available by the authors, without undue reservation, to any qualified researcher.

## Ethics Statement

The protocol was approved by the University of Texas at Austin’s Institutional Review Board and complied with all applicable federal and NIH guidelines. All subjects gave written informed consent in accordance with the Declaration of Helsinki.

## Author Contributions

EH, DB, and FG-L designed the experiment. EH, DB, CS, and PO’C collected the data. DB and FG-L performed the statistical analysis. EH, DB, HL, and FG-L interpreted the results. EH wrote the first draft of the manuscript. DB prepared the figures. All authors contributed to the manuscript revision, read, and approved the submitted version.

## Conflict of Interest

The authors declare that the research was conducted in the absence of any commercial or financial relationships that could be construed as a potential conflict of interest.
